# One year follow-up of the colon cancer patient cohort treated with a novel miniaturized robotic-assisted surgery device (mRASD)

**DOI:** 10.1007/s00464-024-11179-x

**Published:** 2024-09-13

**Authors:** John H. Marks, Michael A. Jobst, Deborah S. Keller, Jorge A. Lagares-Garcia, Henry P. Schoonyoung, Shane M. Farritor, Dmitry Oleynikov

**Affiliations:** 1 Lankenau Medical Center and Lankenau Institute for Medical Research, Wynnewood, PA USA; 2 Bryan Medical Center, Surgical Associates P.C, 1001 S. 70th, Ste. 100, Lincoln, NE 68510 USA; 3 Roper St Francis Health Alliance, Charleston, SC USA; 4 Lankenau Medical Center, Wynnewood, PA USA; 5 Department of Mechanical Engineering, University of Nebraska, Lincoln, NE USA; 6 Monmouth Medical Center Robert Wood Johnson and Barnabas Health, Long Branch, NJ USA

**Keywords:** Colon cancer, Colorectal cancer, Robotic surgery, Robotic colorectal surgery

## Abstract

**Background:**

With the proven benefits of minimally invasive surgery, there is steady growth in robotic surgery use and interest in novel robotic platforms. A miniaturized Robotic-Assisted Surgery Device (mRASD) has been in clinical use under a multi-center, investigational device exemption (IDE) study for right and left colectomy. The goal of this work was to report the short-term and 12-month outcomes specifically for the cohort of colon cancer patients that underwent surgery using the mRASD.

**Method:**

From the IDE study that included both benign and malignant diseases, long-term follow-up was only conducted for patients with colon cancer. The main outcome measures were the oncologic quality metrics (Overall Survival, OS and Disease-free Survival, DFS). Secondary outcomes included incidence of intra-operative, device-related, and procedure-related adverse events. Frequency statistics were performed to assess the measures of central tendency and variability in short (within 30 days) and long-term (1-year) outcomes.

**Results:**

Thirty total patients underwent a colectomy with mRASD; 17 (57%) were diagnosed with a malignancy and included in this analysis. The mean patient age was 59.9 ± 13.2 years. There were no intraoperative or device-related adverse events. In 100% of cases (*n* = 17), the primary dissection was completed and hemostasis maintained using the mRASD, and negative margins were achieved. At 30 days postoperatively, the major complication rate was 6%, and there was one unplanned reoperation for anastomotic leak. At one-year follow-up, the OS and DFS rates were 100 and 94%, respectively. In one patient, omental implants were discovered at the time of surgery, and the patient opted to not undergo additional therapy.

**Conclusions:**

The first experience with mRASD for colectomy in colon cancer demonstrated technical effectiveness and an acceptable surgical safety profile in line with other minimally invasive procedures. The study continues to monitor disease recurrence and survival outcomes in this cohort.

Minimally invasive surgery (MIS) has been establishing its position as a standard of care in the treatment of colorectal cancer (CRC), with robotic-assisted surgery (RAS) increasingly adopted as a MIS technique for CRC procedures. In a review of the National Inpatient Sample (NIS) database, the percentage of malignant colectomy hospitalizations utilizing RAS grew with a 400-fold increase from 2008 to 2017 [1]. The NIS database represents over 35 million hospitalizations, and in 2017, most CRC procedures were performed via an open approach. In a study of the ACS-NSQIP database, the overall trend in RAS for elective non-urgent colectomies was 16% in 2018 with a growth rate of 2.5% per year [2]. While MIS procedures are reported to have significantly shorter hospital stays, fewer complications, and lower reoperation and readmission rates than open procedures [3–8], the odds of utilization of a MIS approach (Laparoscopy or RAS) are reported to decrease with patient age, income, and race; hospital size and location; and primary payer [1, 9–11].

A recently approved miniaturized robotic-assisted surgery device (mRASD), the MIRA Surgical System (Virtual Incision Corporation, Nebraska, USA), was designed as a cost-effective alternative solution to traditional mainframe platforms, which have much larger footprints, to expand RAS access to a larger number of patients irrespective the site of care or geography. The short-term safety and feasibility of the device have been previously reported for colon surgeries in an IDE (#G200257) clinical trial [12]. The IDE study was performed in accordance with the IDEAL Framework (Idea, Development, Exploration, Assessment and Long-term follow-up) for implementing surgical innovation into practice and deemed Stage 2b- exploration [13].

In the IDE study, approximately 40% of the preoperative diagnoses were for colon cancer, and the study protocol required long-term follow-up, up to five years, of all preoperative malignancy cases as well as those determined during surgery.

The aim of this study was to report on the perioperative outcomes and 12-month survivability (disease-free survival and overall survival) in the cohort of cancer patients who underwent a colectomy with the mRASD system.

## Methods

A prospective, multicenter, IDE (#G200257; Clinicaltrials.gov identifier NCT04703829) clinical trial was approved by the FDA to evaluate the safety and effectiveness of the mRASD system in colectomy procedures. The single-arm study was performed at three (3) sites with 6 investigators in the United States. Patients were eligible for enrollment if they had a malignant or benign indication for colectomy and were recruited either through physician referral to a mRASD-trained surgeon or the trained surgeon’s practice.

Thirty patients (30) provided their written, informed consent and enrolled in the study from August 2021 to February 2023, and Investigational Review Board approval was obtained at all study sites. Subjects were evaluated pre-, intra-, peri-, and postoperatively at 30-days. The FDA-approved primary endpoints were the successful completion of pre-defined procedural steps without conversion to open surgery and the overall intraoperative and postoperative adverse events (AE), as well as the incidence of serious AEs (device and/or procedure-related) 30 days postoperatively. All adverse events were captured and adjudicated as related to the device or procedure by an independent Clinical Events Committee (CEC) and Data Safety Monitoring Board (DSMB). In addition, adverse events were also analyzed per the Clavien-Dindo classification system [14].

Long-term, 12-month follow-up was performed in patients who had known colon cancer preoperatively, and those who were diagnosed with cancer intraoperatively or perioperatively. Such patients were required, per the clinical protocol, to have postoperative follow-up at 12, 24, 36, 48, and 60 months. The incidence of cancer status (e.g., active, remission, metastasized, reoccurrence) and incidence of additional surgery/procedures to treat pre- existing cancer at time of procedure were assessed during the follow-up interval.

### Device description

The miniaturized robotic-assisted surgery device (mRASD) differentiates itself from currently available RAS systems in its small formfactor, which translates into a portable system that can be set up at any operating table by means of a sterilizable support arm and controlled from the surgeon console. Designed to be convenient, capable, and compact, it can be set up in any operating room in minutes—without needing to drape or dock. This makes any operating room RAS-ready; allowing more patients to potentially benefit from a minimally invasive approach. The device is indicated for the mobilization of the colon in adults, at least 5′0″ (1.52 m) tall and having a weight of at least 100 lbs. (45.36 kg), who are undergoing minimally invasive colectomy procedures.

It is intended to assist in the visualization of tissues and provide accurate and precise control of surgical instruments to grasp, retract, and dissect while maintaining hemostasis with electrocautery during manipulation of tissues.

**Fig. 1:**
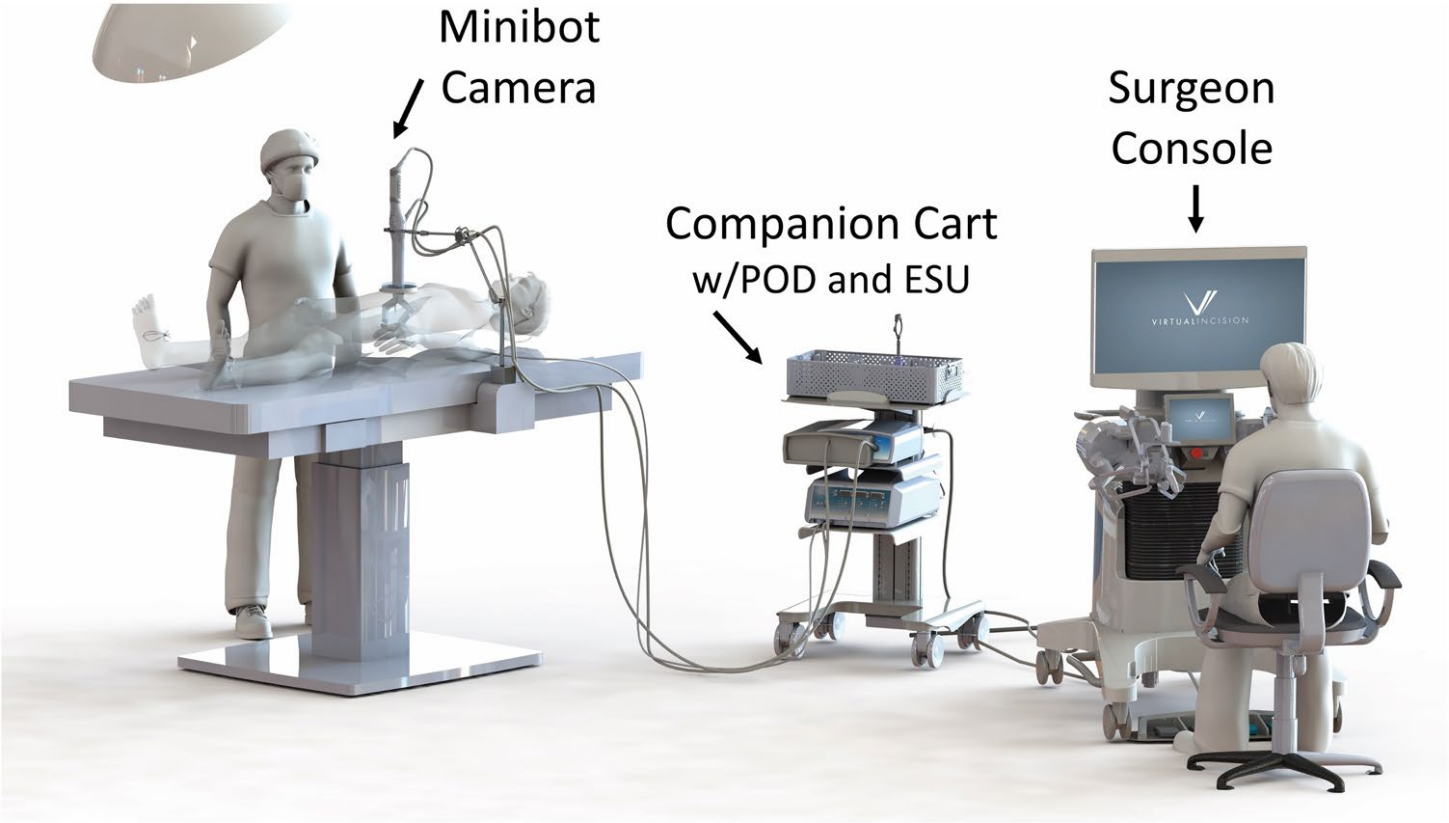
The mRASD surgical system: surgical Minibot and articulated camera, companion cart (with the electrical surgical unit), and surgeon console

The system consists of an integrated Minibot and camera, an open-concept surgeon console, and a companion cart (see Fig. 1). mRASD is designed to be used with the GelPort® Laparoscopic System (Applied Medical Technology Inc, Brecksville, Ohio). The Minibot and camera are inserted into the patient’s abdominal cavity through the insertion port and are remotely controlled via the Surgeon Console. The surgeon console includes a main display showing the real-time video feed from the camera, hand input devices, pedal inputs, and an interactive touchscreen.

### Surgical technique

The hand-assist port was placed in the peri-umbilical midline or through an off-midline, muscle splitting incision at the surgeon’s discretion. The abdomen was insufflated up to 15 mmHg. A 12 mm trocar was placed under direct visualization, 10–12 cm away from the port; in the right lower quadrant for a left/ sigmoid colectomy and along the left costal margin for a right colectomy. The mRASD was deployed through the GelPort under direct visualization. The Minibot arms were deployed inside the abdomen and oriented towards the target anatomy. A medial to lateral or lateral to medial dissection was performed using robotic manipulations with the bipolar grasper and monopolar scissors. Major vessels were divided using a laparoscopic vessel sealer, and bowel was transected using a laparoscopic linear stapler. In left colon resection, the anastomosis was accomplished in a clinically standard manner. Following the Minibot extraction, the specimen was also extracted through the same site. An EEA anvil head was placed, and the colon and anvil head were returned to the peritoneal cavity. Pneumoperitoneum was reestablished, and anastomosis was performed under laparoscopic visualization using the robotic camera. For right colon resection, the anastomosis was accomplished extracorporeally by a functional end-to-end or side-to-side stapled or sutured anastomosis in a standard fashion.

### Statistical methods

Descriptive statistics were performed for categorical variables and results are presented as absolute numbers and percentages or mean and standard deviations.

## Results

Seventeen (17) patients were in the cancer cohort, 13 with a preoperative diagnosis and four with a perioperative diagnosis of colon cancer. Indications for surgery for those four patients were tubulovillous adenoma, large colon polyp, and mass. Three patients (18%) had an additional history of cancer other than colon (1 prostate and 2 melanoma). A majority (59%) of the patients were male with a left colectomy (LC) performed in 10 of the patients and a right colectomy (RC) in seven patients (Table 1). The mean operating time was 161.1 ± 61.5 min, and the mean length of hospital stay was 4.5 ± 8.1 days.

**Table 1:** Patient demographics, operative details, and pathology outcomes

Age, mean (SD), years	59.9(13.2)
Sex, Female (%)	41
BMI (kg/m^2^), mean (SD)	26.8(5.7)
BMI Classification^a^ *n* (%)	
Underweight or normal, 0–24.9	7(41)
Overweight, 25.0–29.9	7(41)
Obese, ≥ 30.0	3 (18)
ASA grade^b^, *n* (%)	
Grade I	0(0)
Grade II	11(65)
Grade III	6(35)
Prior abdominal surgery, No. (%)	10(59)
Intended operation, No. (%)	
Left Colectomy	10(59)
Right Colectomy	7(41)
Preoperative Diagnosis, No. (%)	
Known Cancer	13(76)
Tubulovillous adenoma	2(12)
Cecal Polyp	1(6)
Sigmoid Polyp	1(6)
Operating time, Mean (SD), minutes	
Overall	161.1(61.5)
Left Colectomy	180.0(69.7)
Right Colectomy	134.1(36.7)
Console time, Mean (SD), minutes	89.9(36.8)
Length of Stay, Mean (SD), days (range)	4.5(8.1) (2–36 days)
Pathology Outcomes	
TNM stage, No. (%)	
0	2(12)
1	8(47)
2	1(6)
3	5(29)
4	1(6)
Lymph Node Yield, Mean (range)	26(10–64)
Lymph Node Yield < 12, No. (%)	2(12)
Lymph Node Status, No. (%)	
Positive	4(24)
Negative	13(76)

^a^Derived from World Health Organization classification of obesity based on BMI, with a BMI of 25.0 to 29.9 indicating overweight and a BMI of 30.0 or greater indicating obese

^b^ASA IV subjects excluded from the IDE Clinical Study

### Perioperative and 30-day outcomes

In all cases, the primary dissection was completed and hemostasis maintained using the mRASD. The mean number of harvested lymph nodes retrieved at pathology was 25.8 ± 14.4, and the lymph node yield was > 12 in 88% of the patients. Lymph nodes were positive for metastasis in 4 of 17 (24%) patients. Of the 17 patients who had an operation, 15 (88%) had complete pathology data on margin status available, and all had negative margins. For the other two patients, notes indicated that no residual invasive carcinoma or dysplasia were found following endoscopic excision.

The incidence rates of intraoperative device and procedure-related adverse events were 0%, and a conversion to open surgery occurred in none of the patients (0% conversion rate). The overall 30-day morbidity was 29%, with five patients reporting a complication (see Table 2). Complications included ileus, blood loss anemia, and wound dehiscence. All major complications (Clavien-Dindo Grade > 2) were observed in a single patient (5.9%). Three (3) Grade III complications and 1 Grade IV complication occurred in the patient. One such complication was an anastomotic leak that ultimately required a transfusion and reoperation for the patient, which was the only occurrence of a reoperation in the IDE study.

**Table 2:** Perioperative and 30-day outcomes

Perioperative and 30-day outcomes	
Intraoperative Adverse Event, No. (%)	0 (0)
Postoperative Adverse Event within 30 days, No. (%)	5(29)
Major Complication (Clavien-Dindo > 2) within 30 days, No. (%)	1 (6)
Mortality within 30 days, postoperatively, No. (%)	0 (0)

### 12-month outcomes

No deaths occurred in the cohort at both 30 days and 12 months postoperatively, for a 100% overall survival rate. At 12 months, the remission rate (Disease-free survival rate) was 94% (16 of 17 patients) (Table 3). Cancer was active in one patient (6%). Local recurrence on endoscopy was not observed in the group, and there were no reports of distant metastases. For the one patient with an active cancer, omental implants were discovered at the time of surgery. The LNY was 20 for that patient and the nodes were positive. The patient was determined to be a TNM Stage 4 and is currently not receiving systemic therapy.

**Table 3:** A 12-month outcomes

12- month outcomes (cancer status)	
Incidence of Active, Receiving treatment	0 (0)
Incidence of Active, not-receiving treatment, No. (%)	1 (6)
Incidence of Remission, No. (%)	16 (94)
Incidence of Local Reoccurrence, No. (%)	0 (0)
Incidence of Distal Metastases, No. (%)	0 (0)
Mortality No. (%)	0 (0)

## Discussion and conclusions

This study provides evidence that the mRASD system was effective in complex, high risk patients. In the IDE study, 57% of the patients (17 of 30) had colon cancer, and among that group, 35% were ASA III and 47% were ≥ 65 years of age. The 30-day morbidity was 29% in the cancer cohort with a complication rate of 6% for Clavien-Dindo Grade > 2. At 12 months follow-up, 94% of the patients were in remission, and the overall survival rate was 100%.

Colectomy remains the main therapeutic strategy in the management of colon cancer, and our results, along with the published outcomes of the MIS approach (RAS and Laparoscopy), suggest that the clinical benefits of RAS are maintained through long-term follow-up. In a matched dataset using the ACS-NSQIP (2015–2020) database, Farah et al. reported that both the conversion rate to open and the length of hospital stay were significantly less for RAS patients than laparoscopy, while the complication rate (Clavien-Dindo grade > 2) was 6.0% in both groups [15]. Also, they found that more lymph nodes were harvested with the RAS approach [15]. Similar findings were reported by Rein et al. utilizing data from the Danish Colorectal Cancer Group database (during the period from 2014 to 2019) [16]. In their study, robotic left colectomies were compared to laparoscopic left colectomies. Rein et al. observed a significantly higher proportion of patients with a LNY ≥ 12 in the RAS group than the laparoscopic group, and they found no difference in the postoperative complication rates [16].

Despite the observed short-term benefits of minimally invasive surgery, there is limited long-term high-level evidence supporting its use over traditional approaches in cancer surgery. Nelson et al. compared outcomes between laparoscopy and open surgery for colon cancer in their randomized controlled study. The authors reported a shorter hospital stay and narcotic and analgesics usage in the MIS group, and a similar overall complication rate between the groups [6]. At 3 years, there was no significant difference in the recurrence rate between the groups, and overall survival and disease-free survival rates were comparable also [6]. A randomized, controlled study by Park et al. evaluated the short-term and long-term outcomes between RAS and laparoscopy in patients with right-sided colonic cancer [17]. No significant differences in length of hospital stay, surgical complications, postoperative pain scores, or number of lymph nodes harvested were found between the groups [17]. The 3-year and 5-year overall survival (OS) and disease-free survival (DFS) rates were comparable between the groups also [18].

In a retrospective study of patients who underwent RAS and laparoscopic hemicolectomies for left‑sided colon cancers, Xu et al. reported on the long‑term outcomes of RAS patients. At 3 years, the authors found a disease-free survival rate of 89.8% in the RAS group and 89.0% in the laparoscopic group [19]. Utilizing data from a patient registry (the UICC—Union for International Cancer Control), Cuk et al. evaluated the long-term survival in patients undergoing RAS and laparoscopy surgery for colon cancer. At around 4.93 years, the authors found that patients undergoing RAS had a significantly decreased risk of cancer recurrence (12.4% vs 17.1, *p* = 0.002) [20]. Using data from the National Cancer Database (2013–2019), Emile et al. reported that RAS colectomy was associated with longer overall 5-year survival compared to laparoscopy for certain patients, such as women, patients with a Charlson score of 0, stage II–III disease or left-sided cancer [21]. Likewise, in their meta-analysis, focusing on the long-term outcomes in patients who underwent RAS and laparoscopic right hemicolectomies for colon cancer, Kim et al. found no significant differences in disease-free survival and overall- survival rates between the groups [22]. In another study of the National Cancer Database (2010–2018), Pancheo et al. evaluated the short- and long-term outcomes between RAS and laparoscopic colectomy in elderly patients (≥ 80 years old) diagnosed with colon cancer. In that study, the authors reported a significantly shorter LOS for RAS patients (6.3 vs 5.8 days, *p* < 0.001), and a higher median overall survival rate for RAS patients [23].

Our study has several limitations relating to the small number of patients and study design, as long-term outcomes were not primary endpoints in the initial IDE clinical study. The aim of the IDE study was not to establish superiority or non-inferiority to current surgical approaches, and in our study, we report on the 12-month oncological status and survival of the cohort from the IDE study. While there have been few studies evaluating the long-term oncological outcomes of RAS compared to conventional approaches, our data are consistent with the evidence in the published data for RAS patients.
